# [Retracted] Cocktail therapy with a combination of interferon, ribavirin and angiotensin-II type 1 receptor blocker attenuates murine liver fibrosis development

**DOI:** 10.3892/ijmm.2026.5862

**Published:** 2026-05-18

**Authors:** Hitoshi Yoshiji, Ryuichi Noguchi, Yasuhide Ikenaka, Kosuke Kaji, Yosuke Aihara, Yusaku Shirai, Junichi Yoshii, Koji Yanase, Hiroshi Fukui

Int J Mol Med 28: 81-88, 2011; DOI: 10.3892/ijmm.2011.658

Following the publication of the above article, a concerned reader drew the Editor's attention to the fact that, regarding the microphotographs shown in Fig. 1, panels B and C, and E, F and G contained overlapping sections, where different treatment groups for the respective figure parts were reported in the figure legend. In addition, for the immunohistochemical images shown in Fig. 3, panels A-C also contained overlapping sections; moreover, Fig. 3A, C, F and H contained a series of internally duplicated groupings/repeated patternings of cells within these panels that could not easily be attributed to coincidence. Finally, concerning the tube formation experiments shown in Fig. 7, the 'ARB', 'Rib', 'IFN+Rib' and 'ARB+Rib' panels all apparently contained overlapping sections.

After having conducted an internal investigation of the data in this paper, the Editor of *International Journal of Molecular Medicine* has decided that this article should be retracted from the publication on the grounds of an overall lack of confidence in the presented data. The authors were asked for an explanation to account for these concerns, but the Editorial Office did not receive a reply. The Editor sincerely apologizes to the readership for any incovenience caused, and we thank the reader for drawing this matter to our attention.

